# Health Tract, No. 268

**Published:** 1867-03

**Authors:** 


### ADULTERATIONS.

THE times and principles of men are so out of joint that when we sit down to a table and suppose we are eating a particular dish the chances are ten to one that we are not eating that at all but are eating something else, unless we are partaking of some native product of which we know everything; such as our own vegetables, fruits and fresh meats. Eggs have not yet been counterfeited, but as to milk where is any in our large cities that is not a mixture? A hundred mixtures make our ground coffee, and our tea is made over after it has been used at the tables of hotels. There is a substance called terra alba, or white earth, brought from Ireland for two and a half cents a pound which enters largely into many of our confections; and when sugar costs from fifteen to twenty cents a pound, the temptation to adulterate is scarcely to be resisted by unprincipled shopkeepers. The body of candies and the coating of lozenges and almonds are made of this in many cases, as it is whiter than plaster, and is largely used in the adulteration of flour. In one ounce of lozenges two-thirds of the weight, when dissolved in water, was nothing but this white earth, and the lozenge did not contain an atom of sugar of any kind. Gum Arabic is too costly for pure gum drops to be made to advantage, so a substitute is made, which, although it is beautiful to look at, is very poisonous.

Liqourice drops are made for the trade of the poorest kind of sugar and lamp-black, and merely flavored with liqourice. Twenty parts of liqourice and eighty per cent. of white earth is dextriously mixed and sent to the South and West as pure liqourice. Traders do not hesitate to use the most virulent poisons to make pickles appear fresh and green; while it is a notorious fact that skilled persons can by a combination of drugs make almost any liqour known, and which will so nearly resemble the taste of the true article that experts are deceived. To escape the impositions, it is not sufficient that a man have the utmost confidence in his grocer for he, too, may be profoundly deceived. Let every family have the courage to make its own bread, to prepare its vinegar, to brew its own beer and express its own wines, if they must be had; to buy its own coffee in its green state; to put away its own pickles; to prepare its own sweetmeats, and as to every compound article of food which comes to the table, let it do its own mixing.

